# Nuclear and Cytoplasmatic Quantification of Unconjugated, Label-Free Locked Nucleic Acid Oligonucleotides

**DOI:** 10.1089/nat.2019.0810

**Published:** 2020-01-28

**Authors:** Hannah Pendergraff, Steffen Schmidt, Jonas Vikeså, Christian Weile, Charlotte Øverup, Marie W. Lindholm, Troels Koch

**Affiliations:** Roche Pharma Research and Early Development, RNA Therapeutics Research, Roche Innovation Center Copenhagen, Hørsholm, Denmark.

**Keywords:** LNA, quantification, biodistribution

## Abstract

Methods for the quantification of antisense oligonucleotides (AONs) provide insightful information on biodistribution and intracellular trafficking. However, the established methods have not provided information on the absolute number of molecules in subcellular compartments or about how many AONs are needed for target gene reduction for unconjugated AONs. We have developed a new method for nuclear AON quantification that enables us to determine the absolute number of AONs per nucleus without relying on AON conjugates such as fluorophores that may alter AON distribution. This study describes an alternative and label-free method using subcellular fractionation, nucleus counting, and locked nucleic acid (LNA) sandwich enzyme-linked immunosorbent assay to quantify absolute numbers of oligonucleotides in nuclei. Our findings show compound variability (diversity) by which 247,000–693,000 LNAs/nuclei results in similar target reduction for different compounds. This method can be applied to any antisense drug discovery platform providing information on specific and clinically relevant AONs. Finally, this method can directly compare nuclear entry of AON with target gene knockdown for any compound design and nucleobase sequence, gene target, and phosphorothioate stereochemistry.

## Introduction

The ability of organisms to regulate gene expression is essential for survival. Organisms have evolved to contain endogenous mechanisms that allow the cells to regulate RNAs and proteins from transcription to post-translational levels [1,2]. Antisense oligonucleotides (AONs) are short nucleic acids that are frequently used to inhibit or activate the expression of genes of interest [3–5].

After the first report of antisense gene silencing in 1978 [6], hundreds of potential oligonucleotide drugs have been tested in clinical trials for treatment of many different diseases, including spinal muscular atrophy and Duchenne muscular dystrophy [7–9]. In 2016, Spinraza, a chemically modified AON, was approved to treat spinal muscular atrophy, becoming the only Food and Drug Administration approved treatment for this disease [10].

Once delivered to a cell or organism, the AON must migrate to its complementary target sequence “unassisted” because of the lack of endogenous cellular machinery to protect, guide, or traffic the oligonucleotide to the target [11]. Unmodified synthetic DNA and RNA are sensitive to nuclease digestion, and chemical modifications are incorporated into the backbone of the AONs to inhibit nuclease digestion, aid in biodistribution, and to increase the affinity and potency of an AON to its desired target.

**FIG. 1.**
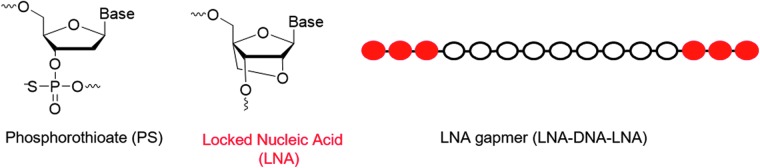
Molecular structures and chemical modifications used in this work.

There are numerous chemical modifications of the oligonucleotide backbone [12], but in this study we only used fully phosphorothiolated (PS) locked nucleic acid (LNA) oligonucleotides (Fig. 1) [13–21]. Synthesis of PS internucleoside linkages is not stereo controlled, and therefore two stereochemical configurations Rp and Sp are created at each coupling. Consequently, all LNA oligonucleotides consist of diastereoisomer mixtures (ie, 2^n^, *n* = PS internucleoside linkages) [22].

Single-stranded AONs can bind to and interact with many different target classes. AONs can inhibit miRNA expression [23–25], induce alternative splicing [26–31], and inhibit mRNA expression by both a steric block [32,33], and an RNase recruiting mechanism [34,35]. Except for miRNA inhibition, it has been shown that AON activity is most effective in the nucleus [36], and in applications involving splice modulation, nuclear entry of AONs is crucial. Because AONs are predominantly taken up by cells through endocytosis [37].

It is an ongoing challenge for AON therapeutics to design compounds that escape the endosomes and efficiently enter the nucleus. There have been many reports on the cellular distribution of single-stranded oligonucleotides [38,39], but the experiments cannot always be directly correlated to therapeutic applications or *in vivo* potential [38].

At present, most studies on intracellular presence and trafficking rely on covalent attachment of a fluorophore onto the oligonucleotide [38–43]. These studies involving fluorescently labeled oligonucleotides are beneficial to understanding the distribution, and some constructs mimic very well the situation of unconjugated AON [44]. However, chemically modified AONs will not, or cannot, act exactly *in vitro* and *in vivo* as the unconjugated AON, and some reports have shown that certain modifications or fluorescent tags alter the properties of an oligonucleotide significantly [45–48].

Other methods that are used to quantify AONs are capillary gel electrophoresis and liquid chromatography–mass spectrometry (LC-MS) [49]. Although the methods are quantitative, they require extensive optimization for purification of the full-length AON from the metabolites in biological samples [50–52]. However, as reported by Erb *et al.* [53] the limit of detection for AONs is in the nanomolar range that may exclude nuclear retained oligonucleotides from the analysis. LC-MS is an important tool for whole-cell or whole-tissue quantification, but our method may be applied to much smaller quantities of AONs. Furthermore, as our methods require LNA capture probes to target a full-length AON, any potential AON metabolites will not be captured and thus, included in the quantification.

We developed a novel quantification method for unlabeled AONs in the nucleus of cells. Using this method, we determined LNA content in the nuclei for several molecules and correlated the data to target gene knockdown for different gene targets. In a concentration–response experiment, we showed that the number of LNAs per nucleus, after a minimum detection threshold is met, is proportional to target transcript reduction. However, comparing across several targets and compounds we demonstrate that a high nuclear concentration alone is not the only determinant for strong target transcript level reduction.

## Materials and Methods

### Synthesis of LNA gapmers and enzyme-linked immunosorbent assay probes

Oligonucleotides were synthesized on a MerMade192 instrument in 1 μmol scale on UnyLinker controlled pore glass (CPG) solid support using phosphoramidite monomers and a synthesis cycle consisting of detritylation, coupling, sulfurization or oxidation, and capping. Three percent trichloroacetic acid in dichloromethane was used for detritylation, 0.25 M dicyanoimidazole in acetonitrile was used as activator, 0.1 M xanthane hydride in 1:1 pyridine and acetonitrile was used for sulfurization, tetrahydrofurane/water/pyridine/iodine 90.54/9.05/0.41/0.43 (v/v/v/w) was used for oxidation, acetic anhydride/tetrahydrofurane 9.1/90.9 (v/v) was used as CapA and tetrahydrofurane/*N*-methylimidazole/pyridine 8/1/1 (v/v/v) was used as CapB. After completion of solid-phase synthesis, the oligonucleotide was cleaved from the support and deprotected by suspending the solid support in concentrated aqueous ammonia at 55°C for 4 h. Unconjugated oligonucleotides were synthesized 4,4′-dimethoxytrityl group ON (DMT-ON) and purified by reversed-phase solid-phase extraction on Agilent TopDNA cartridges. 5′-Digoxigenin (DIG) labels were synthesized by conjugation of DIG N-hydroxysuccinimide (NHS) ester (Sigma Aldrich) to aminododecyl-modified oligonucleotides as described hereunder. The aminododecyl label was introduced during solid-phase synthesis as a phosphoramidite (5′-TFA-Amino-Modifier-C12-CE Phosphoramidite from Link Technologies) in the final coupling cycle. 3′-Biotin labels were synthesized by conjugation of Biotin NHS ester (Sigma Aldrich) to amino C6-modified oligonucleotides as described hereunder. The amino C6 label was introduced during solid-phase synthesis as a solid support (3′-Amino Modifier TFA Amino C-6 lcaa CPG 1000 Å from ChemGenes). Conjugation of NHS esters to amino-modified oligonucleotides: After cleavage and deprotection of the aminohexyl oligonucleotide, the ammonia was removed *in vacuo*, and the oligonucleotide was dissolved in 1 mL water, and filtered through a 0.45 μm syringe filter. Thereafter the aminohexyl-labeled oligonucleotides were precipitated as lithium salt by addition of 5 mL 2% (w/v) LiClO_4_ in acetone to prepare for conjugation. The precipitate was recovered by centrifugation, and the supernatant was decanted. The resulting oligonucleotide pellet was dissolved in 200 μL, 100 mM sodium carbonate buffer pH 8.5. To this solution was added 2.5 mg of the respective NHS ester in 75 μL anhydrous dimethyl sulfoxide. The conjugation proceeded overnight. Thereafter the product was precipitated from the solution by addition of 1 mL 2% (w/V) LiClO_4_ in acetone. The precipitate was recovered by centrifugation, and redissolved in 1 mL MilliQ water filtered through a 0.45 μm syringe filter. The conjugates were purified by preparative reversed-phase high-performance liquid chromatography on a Jupiter C18 column with a 5%–60% acetonitrile gradient in 0.1 M ammonium acetate pH 8 in MilliQ water over 15 min with a flow rate of 5 mL/min. Fractions were collected based on absorption at 260 nm. The fractions containing desired product were concentrated *in vacuo* and dissolved in phosphate-buffered saline (PBS) buffer. All products were analyzed by ultra performance liquid chromatography (UPLC)-MS to confirm identity and purity. Threshold limit for inclusion of *n* − 1 products must fall within 0.05% ± the calculated molecular weight by mass spectrometry, and must be >85.0% pure by UPLC based on the percent area at 260 nm.

### Cell culture and gymnosis

LNAs were synthesized in house and reconstituted in either nuclease-free water or 1 × phosphate-buffered saline (PBS). HeLa cells were obtained from European Collection of Authenticated Cell Cultures (ECACC). HeLa cells were maintained at 37°C at 5% CO_2_. Minimal essential media was supplemented with 10% fetal bovine serum, 0.5% glutamine, 0.5% nonessential amino acids, and 250 μL gentamicin (all from Sigma). Approximately 1.8 million cells were seeded in a T-75 cell culture flask with LNAs at a documented concentration in 12 mL of full culture media. The cells grew to confluency in LNA supplemented media for 72 h before being harvested for experiments. After the 72-h incubation, cells were washed with 1 × Dulbecco's phosphate-buffered saline (DPBS) (Sigma), trypsinized with 1.5 mL of 0.25% trypsin (Thermo Fisher), incubated at 37°C at 5% CO_2_ for 5 min, then an additional 10.5 mL of media was added to the flask. Cells were then counted using a Chemometec NucleoCounter NC-200 according to manufacturer's instructions. A 500 μL aliquot was taken from each sample for whole cell and whole enzyme-linked immunosorbent assay (ELISA) content analysis. The remaining cells were used in further subcellular fractionation experiments.

### Subcellular fractionation

After cell counting, an equal number of HeLa cells were used for each sample in an experiment. For instance, all samples had 5 million cells. The fractionation modified previously published protocols [54]. The cells were spun at 500*g* for 5 min at 4°C. The supernatant was discarded, and the cells were washed with ice-cold 1 × DPBS (Sigma) before being centrifuged for another 5 min at 500*g* at 4°C. The 1 × DPBS was then aspirated and the cell pellets were resuspended in hypotonic lysis buffer (HLB) [10 mM Tris, pH 7.5, 10 mM NaCl, 3 mM MgCl_2_ (all from Ambion), 0.3% NP-40 (v/v; Sigma), and 10% glycerol (v/v; Sigma)] supplemented with 1 × protease inhibitors (Thermo Fisher) and phosphatase inhibitor solution (100 × concentration contains 0.1 M sodium orthovanadate and 0.1 M sodium fluoride (both from Sigma) in nuclease-free water, incubated on ice for 10 min, vortexed gently, and centrifuged at 800*g* for 8 min at 4°C. The supernatant was collected as the cytoplasmic fraction and transferred to a clean 1.5 mL Eppendorf tube. Fifty-six microliters of 5 M sodium acetate (Ambion) was added to each cytoplasmic sample. For the nuclear washing control experiment, the nucleus of nontreated (NT) cells was resuspended in the cytoplasm of cells treated with LNA1, incubated for 10 min, and vortexed before proceeding to the following steps. The remaining nuclear pellets were washed with 500 μL of HLB supplemented with 1 × protease inhibitors and phosphatase inhibitor solution then centrifuged at 200*g* for 2 min at 4°C. This wash step was repeated three additional times. The nuclei were resuspended in 800 μL of 1 × PBS and sorted through a BioRad Se3 cell sorter by size (Supplementary Fig. S1) to only proceed with intact nuclei. The quantity of nuclei sorted was recorded for future calculations. After sorting, 500 μL of nuclear lysis buffer [20 mM Tris, pH 7.5, 150 mM KCl, 3 mM MgCl_2_, 0.3% NP-40 (v/v), and 10% glycerol (v/v)] was added to each sample immediately before homogenization with a Dounce Homogenizer (Sigma). All nuclear and cytoplasmic samples were then spun at 18,000*g* for 15 min at 4°C. The supernatants from the samples were transferred to new two Eppendorf tubes and stored in the −80°C before further analysis.

### Protein analysis

Cytoplasmic protein concentration was calculated using a NanoDrop (Thermo Scientific Nanodrop 1000). Approximately 0.2 mg/mL of cytoplasmic samples were added to the WES ProteinSimple for protein analysis as per manufacturer instructions. For nuclear samples, 4 μL was added per well of the WES ProteinSimple for protein analysis. For the cytoplasmic spike-in experiments, cytoplasmic samples were diluted to a final concentration of 1 mg/mL before being added by volume to a nuclear fraction to a total of 20 μL. We used Lamin A/C antibody 1:25 (NBP2-19324; New England Biolabs) for the nuclear marker, Rab4 1:50 (PA3-912; Thermo Fisher) as the endosomal marker, and tubulin 1:25 (NB500-333; New England Biolabs) as the cytoplasmic marker. Analysis was performed on Compass for SW software.

### RNA preparation

After protein analysis, the samples were concentrated to an approximate volume of 200 μL using heat and air. RNA precipitation was carried out by adding a 1:1 volume of phenol:chloroform solution (Sigma) to the samples, followed by vigorous shaking for 1 min. The samples settled at room temperature for 10 min before centrifuging at 13,000 rpm for 20 min. The aqueous phase was transferred to a new Eppendorf tube. A 1:10 volume of 3 M NaAc (Ambion) and a 2.5 volume of cold 100% EtOH (Sigma) was added to the samples. The samples were put in either −80°C for 20 min or −20°C for several hours. The samples were then spun at 13,000 rpm for 15 min at 4°C. The supernatant was discarded, and the RNA pellet was washed with ice-cold 70% EtOH. The samples were centrifuged at 8,000 rpm for 10 min at 4°C. The supernatant was discarded, and the RNA pellet dried. The pellet was resuspended in 50 μL of RNase-free water (Gibco). The samples were heated at 55°C for 5 min, and then RNase treated by adding 1 U of RNase A/C (Thermo Fisher) per manufacturer's instructions.

### Enzyme-linked immunosorbent assay

LNA quantification from cytosolic and nuclear fractions after precipitation was determined by hybridization dependent ELISA using a biotinylated capture probe and a DIG-conjugated detection probe as described previously [55]. For each LNA a pair of capture and detection probes was designed to hybridize with the AONs. Samples were diluted and incubated with 35 nM biotinylated capture probe and 35 nM DIG-coupled detection probe for 30 min at room temperature in 5 × saline sodium citrate buffer (20 × saline sodium citrate; Sigma) containing 0.05% Tween-30 (Sigma) in a 96-well plate. The assembled complex was then captured on a streptavidin-coated ELISA plate (Nunc) for 1 h and after three washing steps with 2 × SSCT buffer, each well was incubated with an anti DIG-alkaline phosphatase-Fab fragment (Roche) for 1 h at room temperature. After three additional washing steps, Blue Phos Substrate (KPL) was added to the plates and color development was measured spectrophotometrically at 615 nm after 20 min. LNA concentration in the lysate was calculated according to a standard curve generated with the respective stock solution with minimum detection values ranging from 0.03 to 0.35 pmol/mL (Supplementary Table S1). Probes used for this work-all phosphodiester LNAs: LNA1: capture probe TGCTTGC-Bio-3′, detection probe 5′-DIG-ACAGGA; LNA2: capture probe TTATAACT-Bio-3′, detection probe 5′-DIG-AGCTGGA; LNA3: capture probe TAACTC-Bio-3′, detection probe 5′-DIG-TGGCAAG; capture probe LNA4: capture probe CAATAACAA-Bio-3′, detection probe 5′-DIG-CCATCCT; LNA5: capture probe CCAAAG-Bio-3′, detection probe 5′-DIG-TCATTCTG; LNA6; capture probe CTAAGTGA-Bio-3′, detection probe 5′-DIG-ACCAATGG.

### LNA quantification

The LNA concentration in each sample was determined using GraphPad Prism and interpolating the absorbance values from the ELISA to a standard curve of the LNA on each ELISA plate. The results from the ELISA data allowed us to quantify the pmol/mL of LNA in each sample. We then calculated the (fmol/μL) × (volume of sample in μL) to determine the fmol per sample and finally, multiplied by 6.022 × 10^23^ to calculate the number of LNA molecules per sample. The number of LNA molecules was then divided by the number of cells (cytoplasm) or the number of nuclei (counted from a BioRad Se3 cell sorter) per sample. This value results in the number of LNA molecules per cell or nucleus or cytoplasm.

### Quantitative real time-polymerase chain reaction

Approximately 125,000 cells per sample were pelleted, washed with 1 × DPBS, and added to a 96-well quantitative polymerase chain reaction (qPCR) plate. Lysis buffer of 150 μL was added to each sample (Invitrogen Pure Link Lysis buffer). RNA purification was carried out as per manufacturer's instructions. A master mix of One-Step Taqman mix (Thermo Fisher) and Thermo Fisher Taqman assays: *HIF-1α* (Hs00936368_m1), *BCL2* (Hs00608023_m1), *Cers2* (Hs00604577_m1), *Malat1* (Hs00273901_m1), *PTEN* (Hs02621230_s1), and *GAPDH* (4326317E) was made. Six microliters of Taqman mix and 4 μL of RNA were added to a 384-well qPCR plate and analyzed using a Thermo Scientific ViiA7 PCR system with a PCR program of 50°C for 15 min, 95°C for 3 min, followed by 40 cycles of 95°C for 5 s and 60°C for 45 s. Gene knockdown was measured using a ΔΔCT method.

## Results

### Validation of nuclear fraction purity

During our fractionation, great care was taken to avoid any unwanted cytoplasmic contamination in our nuclear fractions. Therefore, the fluorescence-activated cell sorting (FACS) sorter was programmed to only proceed with whole, intact nuclei, as contamination from endosomes could greatly influence our calculations. To ensure that the nuclear fractions were isolated free from endosomal and cytoplasmic contamination, we first used a WES ProteinSimple western blot system [49,56–58] to analyze protein from nuclear and cytoplasmic samples (Supplementary Fig. S2).

However, we went further to test the sensitivity of the western blot system. A “spike-in” experiment was performed where known concentrations of cytoplasmic extracts were added in a concentration-dependent manner to a purified nuclear fraction, using cytoplasmic and nuclear extract from a fractionation experiment starting with 6 million cells per sample. Cytoplasmic samples were diluted to 1 mg/mL before starting protein analysis, representing ∼1,000, 2,000, 4,000, 6,000, and 8,000 cell cytoplasmic fractions in 0.5, 1, 2, 3, and 4 μL spike-in samples, respectively.

**FIG. 2.**
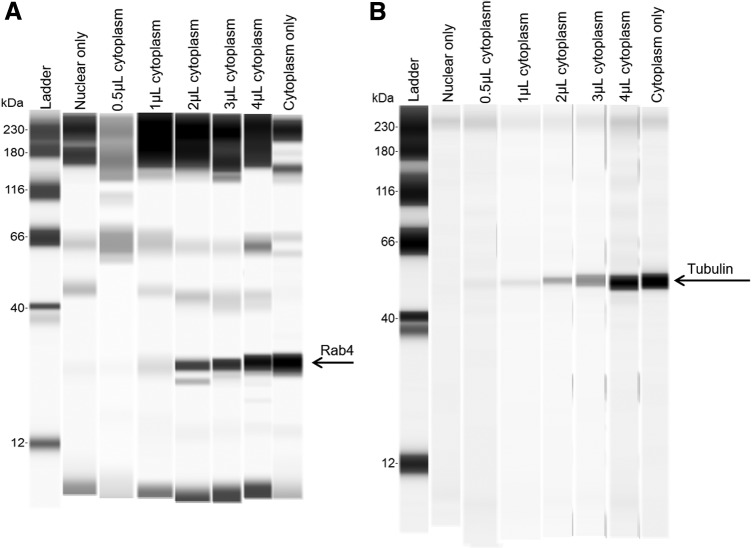
WES ProteinSimple analysis of a spike in experiment using pure nuclear fractions and a dose-dependent volume of cytoplasmic fractions. The data are either **(A)** endosomal Rab4 protein or **(B)** cytoplasmic tubulin protein.

The data show that when there is as little as 0.5 μL of spiked-in cytoplasmic sample (∼2% of the total sample concentration), endosomal or cytoplasmic contamination can be detected in the nuclear fractions (Fig. 2). The spike-in experiments demonstrated that the used method is sensitive enough to detect minute amounts of cytoplasmic contamination in the nuclear fraction. For our experiments, we only proceeded with nuclear samples that had no detectable cytoplasmic or endosomal contamination. From these data, we can be confident that the LNAs detected in our nuclear fractions of our experiments was not a by-product from or contaminated by endosomal or cytoplasmic LNAs.

Furthermore, to ensure that there was no passive diffusion of LNAs to the nucleus from the cytoplasm during the fractionation experiments, wash steps were performed on nuclei from NT cells that did not receive any LNA. We set up an experiment containing an NT sample, and an LNA-treated sample using an initial concentration of 5 μM LNA1. Once the nuclei were isolated, NT nuclei were washed in the cytoplasmic fraction of the LNA1-treated sample, incubated for 10 min as per the protocol, and then proceeded with the nuclear washing steps. This method mimics the actual fractionation protocol and would not only identify if LNA from the cytoplasmic sample could contaminate the nuclear fraction but also identify if LNAs could attach themselves to the nuclear membrane and give a false-positive signal in the ELISA readouts. The nuclei sample from the treated LNA1 cells was used as a positive experimental control.

Our data show that there is negligible contamination from the cytoplasmic LNAs in our nuclear samples through either passive diffusion or direct cytoplasmic contamination. The nuclei in this experiment were not sorted using the cell sorter so the samples are cruder than fractionations for quantification of LNAs in the nucleus. From these data, a small amount of LNA can be detected in the NT sample incubated with LNA1-treated cytoplasm (Supplementary Table S2).

**FIG. 3.**
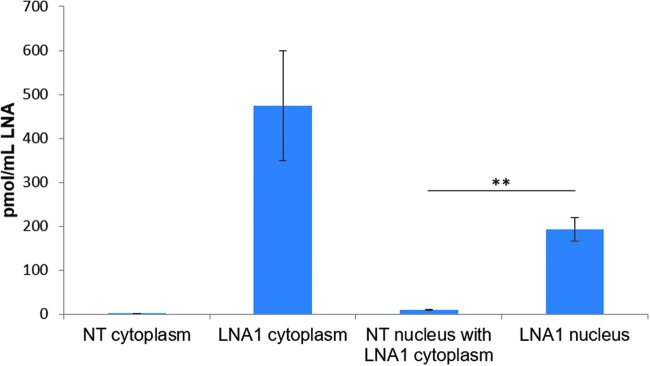
ELISA data from nuclear wash experiment. Nontreated (NT) nuclear samples were incubated in LNA1-treated cytoplasmic cell extract. The data show that there is a 24-fold difference between nuclear LNA content between treated and untreated nuclei. Standard deviation from two independent experiments. **P < 0.01 for nuclear samples. LNA, locked nucleic acid; ELISA, enzyme-linked immunosorbent assay.

However, there is a 24-fold increase in LNA1-treated nuclear sample and the NT nuclear sample (Fig. 3). These data indicate that the nuclear LNA quantifications are the results of LNAs in the nuclear fractions only and there is no passive diffusion of LNAs into the nucleus because of cytoplasmic or endosomal contamination or the fractionation experiment itself.

### Increased nuclear entry is proportional to increased target gene knockdown

**FIG. 4.**
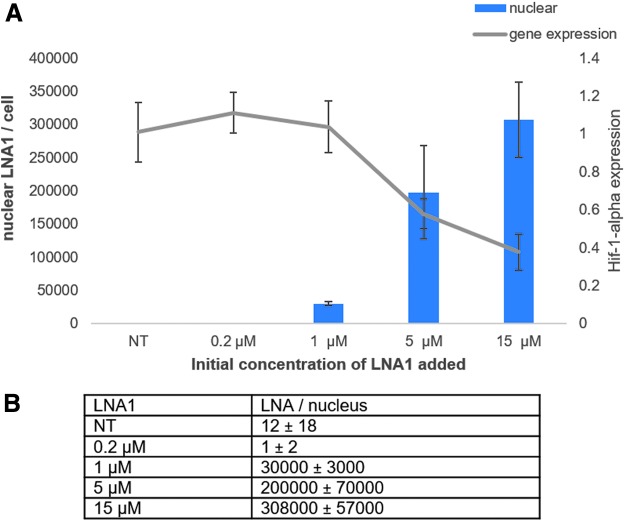
ELISA data showing the calculated number of LNAs in the nucleus of HeLa cells. **(A)** Nuclear numbers of LNAs per cell and the corresponding concentration *Hif-1-α* gene knockdown. All LNA standard deviation values are the values from two independent experiments with two technical replicates each. Error bars for gene expression are standard deviation of six technical replicates. **(B)** Table showing the average quantitated LNA number per nucleus. All numbers are rounded to the nearest 1,000 LNA molecules.

A concentration–response was performed on HeLa cells to determine the relationship between gene knockdown and nuclear LNA AON content using a previously described compound targeted the *HIF-1α* mRNA transcript (Fig. 4) [59]. HeLa cells were treated under gymnotic [60] assay conditions with an increasing concentration of LNA1.

The number of LNA molecules was calculated based on the ELISA data. The readout from the ELISA assay is an absorbance value that allows for a concentration calculation of LNA by interpolation from a standard curve. Using the Avogadro constant along with the volume of sample, we calculated the number of LNA molecules in nuclear samples. The number of molecules per sample was divided by the known number of nuclei (calculated from the cell sorter) in our samples to get the average LNA value per nuclear fraction per cell.

*HIF-1α* target gene reduction correlates with the number of LNA molecules in the nucleus when the nuclear accumulation reaches a threshold of ∼10^5^ molecules per nucleus. The data show that at the lower dose (0.2 μM) we are unable to measure nuclear LNA. Whether this is because of the fact that LNAs are not escaping the nonproductive compartment at this concentration, or the amount of nuclear LNAs is below our limit of detection, is unclear. Not surprisingly, with an undetectable amount of LNA per nucleus at the 0.2 μM concentration, we did not observe any target gene reduction.

We calculated an average of 30,000 LNAs per nucleus at the 1 μM concentration, an amount that also did not produce any significant *HIF-1α* knockdown. Although it was known that a threshold of nuclear LNA must be met to see target transcript reduction, it is surprising that a threshold >10^5^ LNA molecules per nucleus is needed for this compound. However, the data correspond well with recent findings from microinjection experiments by Buntz *et al.* [43] demonstrating that levels in the range of 10^5^ are needed for target gene knockdown. There is only a 1.5-fold difference in the number of nuclear LNAs with the 5 and 15 μM concentrations but it results in a 20% greater inhibition of *HIF-1α* at the higher concentration. These data clearly show that for the *HIF-1α* target gene, the higher nuclear concentration of this LNA, the greater the gene silencing. We expect that this would also be the case for other AONs.

### Absolute number of nuclear LNA gapmers needed for target transcript knockdown varies across compounds and is not directly proportional to gene silencing

**FIG. 5.**
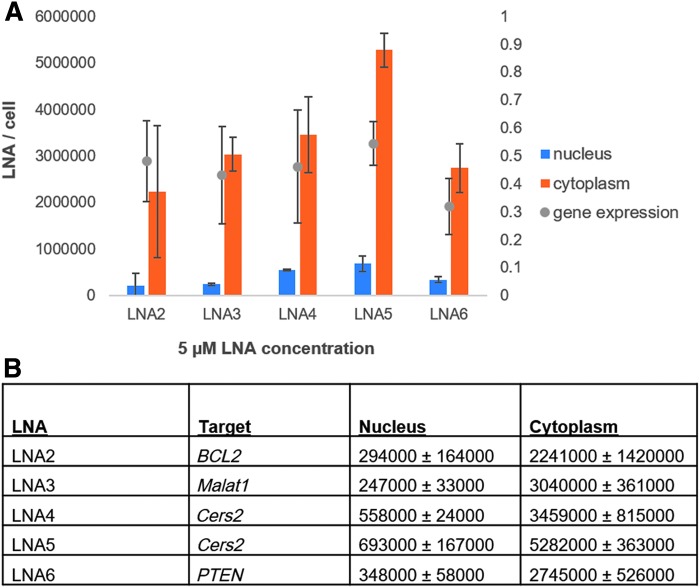
ELISA data showing the calculated number of LNAs in the nucleus and in the cytoplasm of HeLa cells. **(A)** Number of LNAs in the nucleus and in the cytoplasm of HeLa cells in relation to the gene knockdown. **(B)** Calculated values of LNAs in the nucleus and in the cytoplasm based on the ELISA data. Error bars on all experiments are calculated based on two independent experiments. LNA numbers are rounded to the nearest 1,000 molecules.

HeLa cells were treated with a single concentration (5 μM) of five different LNAs, which between them target four different mRNA transcripts (Fig. 5). We chose these additional targets as they all have different cellular functions. The *Cers2* gene plays a role in the regulation of cell growth, *BCL2* encodes an outer mitochondrial membrane protein that can block apoptosis in cancer cells, *PTEN* is a tumor suppressor that can be mutated in cancer cells, and *MALAT1* is a noncoding RNA that is highly expressed in the nucleus.

The five different compounds exhibited a 40%–65% reduction of their respective target transcript under the tested experimental conditions. The nuclear content of the 5 different compounds varied by a factor of three. For these samples, the number of cytoplasmic LNAs was also calculated. We tested two different LNAs against the same target and one LNA, LNA5, had a higher efficiency of uptake compared with LNA4. For these LNAs an amount of 693,000 and 558,000 per nucleus, a target knockdown of 40%, was exhibited. These data indicate that *Cers2* is either a difficult transcript to target or that a concentration of 5 μM is insufficient to achieve effective gene silencing in the gymnotic assay.

We observed that the gene silencing effect of LNA2 and LNA3, targeting *BCL2* and *MALAT1* were both at an average of 50%. However, these two LNAs had lower nuclear LNA quantities than the *Cers2* and *PTEN* targeting LNAs. Both LNA2 and LNA3 had a greater target transcript reduction at 5 μM than LNA1, but LNA1 also had a lower number of LNAs per nucleus. On the contrary, the *Cers2* molecules had a higher quantity of LNAs per nucleus but had a lower target transcript reduction.

The most potent compound measured at the 5 μM concentration was the LNA targeting *PTEN*. This molecule had one of the lowest cytoplasmic quantities with 2.7 million LNAs per cytoplasm at 5 μM but had a quantity of 348,000 per nucleus. Of interest, LNA1 was the only other compound tested that achieved >70% target transcript reduction, but at a higher initial LNA concentration (15 μM) and a lower number of nuclear LNAs per nucleus (308,000).

## Discussion

The relationship between oligonucleotide uptake and efficacy has always been a critical question in oligonucleotide therapeutics. One explanation for the lack of a direct correlation between uptake/activity has been that a large fraction of single-stranded oligonucleotides are getting trapped in the endosomes and are unable to escape to the productive nuclear compartment of the cells. This is one of the reasons why there has been a great interest in developing endosomal escape methods for oligonucleotides, and many designs and conjugates have been tested, including cell-penetrating peptides, proteins, and viral delivery agents [61,62] to aid in AON endosomal release. However, manipulation with endosomal integrity and biology is critical for cellular viability and many of these endosomal escape constructs increased the cellular toxicity significantly [63].

Subcellular fractionation methods have been invaluable tools in the identification of organelles and proteins within a specific cellular compartment [64,65]. Through the ability to isolate different fractions of the cells, there have been many advances in the understanding of protein synthesis mechanisms [66,67] the replication of DNA [68,69], DNA transcription [70], RNA splicing [71], and gene localization [72] to name a few. The ability to isolate nuclear and cytoplasmic compartments shown here has given us an alternative methodology to study AON intracellular trafficking.

The method used for our subcellular fractionation was adapted from a previously published method [53]. Gagnon *et al.* [53] demonstrated clearly that the hyptonic lysis buffer does not lyse the nucleus of the cells. As an extra precaution for nuclear integrity, we employed a cell sorter, which assures that only intact nuclei are isolated and used for analysis. Although redistribution of AONs could be possible during the subcellular fractionation experiment, our data demonstrate that this is unlikely or in the worst case, minimal. In the negative control experiment, we confirmed that there is negligible redistribution of cytoplasmic AONs into the nucleus after the fractionation protocol. Furthermore, a recent publication by Buntz et al [43] shows that AONs that enter the nuclear compartment of the cells do no redistribute back into the cytoplasm. Buntz *et al.* [43] also confirms our finding that a minimum of 10^5^ AONs are needed in the nucleus for gene silencing to occur. In conclusion, because the lysis buffer does not compromise the nuclear fraction, we use a cell sorter that retain nuclear integrity, AONs entering the nucleus are retained, and cytoplasmic AONs do not redistribute to the nucleus during fractionation, we conclude that the values from our experimental results are valid and accurate determinations of nuclear and cytoplasmic content.

The concentration–response data with LNA1 targeting *HIF-1α* shows that a threshold value has to be reached before the target transcript is downregulated. One reason for such a threshold could be that RNA target binding by LNA oligonucleotides is competing with binding to intracellular proteins [59]. Because the latter is in large molar excess compared with the specific RNA targets, protein binding could serve as an oligonucleotide/LNA sponge. Therefore, intracellular protein binding needs to be more or less saturated before excess LNA is available for RNA binding. This mechanism is supported by the fact that only a 1.5-fold increase in nuclear amounts of LNA1 (200,000–308,000 LNAs) exhibited a significant 20% further downregulation (Fig. 4).

Our data show that endosomal escape is not the only factor in determining whether an LNA can achieve potent gene silencing. Most of the tested LNAs did follow a similar pattern where a higher nuclear quantity corresponded to a greater target reduction. *HIF-1α* targeting LNA1 achieved 40% gene inhibition with 200,000 nuclear LNAs, *BLC2* and *Malat1* targeting LNAs both achieved 50% gene inhibition with 294,000 and 247,000 LNAs, respectively, and the *PTEN* targeting LNA with 348,000 molecules in the nucleus achieved 72% gene inhibition. Although these molecules do follow a correlation of higher nuclear LNA quantity, higher activity against a target gene, the *Cers2* molecules did not. Even with almost three times the number of nuclear LNAs as LNA1, we only observed a 40% target transcript reduction. Recently a publication by Gonzalez-Barriga *et al.* [40] reported on the distribution and nuclear concentration of AONs in gymnotic assays. Fluorescently labeled oligonucleotides were used and the quantification results were based on fluorescent intensity in the nucleus. However, their findings are in line with the data reported here showing that the concentration of AONs in the nucleus alone is not adequate for target gene knockdown correlation. Other factors such as protein binding, accessibility of target RNA, and recruitment rate of RNase H will also contribute to AON potency.

Our findings indicate that for some gene targets, and for some compounds as well, potent inhibition will not be reached even if there is a high number of oligonucleotides in the nucleus. In these cases, cytoplasmic, nuclear, and target interaction mechanisms other than endosomal escape may be limiting activity. It is possible that target transcript accessibility is limited in some domains because of chaperone protein interactions or high-affinity structural or tertiary domains. Consequently, if endosomal release is not a limiting factor in all cases, the search for new release agents may be of little value. When this is added to the high risk of cytotoxicity by endosomal release agents, other routes are recommended to be pursued for optimizing AON activity.

We have tested a handful of fully PS LNA gapmers, and further studies will ascertain the correlation and predictive value of the relationship between nuclear AON presence and activity. In particular, it should be explored more comprehensively how different AONs with different gene silencing potencies for the same mRNA target, in different cell lines, correlate with nuclear accumulation. In addition, the role of phosphorothioate stereochemistry should be explored [22,73–76]. We have preliminary data that show a large diversity in a series of single isomer stereo-defined LNA for nuclear accumulation and activity (Pendergraff, unpublished data).

In conclusion, we have shown a label-free and highly sensitive subcellular fractionation of LNA oligonucleotides. A specific assignment of nuclear accumulation is an important step forward for understanding cellular processes and the biomolecular interactions leading to potency and toxicity of AONs. The method represents a direct way to get information about AON drug accumulation with high precision and at the closest to the site of action. Although we have not seen that a specific number of nuclear LNAs correlate with a specific activity across different compounds, nuclear quantification may turn out to be a better basis for developing more predictive activity measures.

## Supplementary Material

Supplemental data

Supplemental data

Supplemental data

Supplemental data

## References

[1] Kohler A and Hurt E (2007). Exporting RNA from the nucleus to the cytoplasm. Nat Rev Mol Cell Biol 8:761–773 17786152 10.1038/nrm2255

[2] Mattick JS, Dinger ME, Mercer TR and Mehler MF (2009). RNA regulation of epigenetic processes. BioEssays 31:51–59 19154003 10.1002/bies.080099

[3] Janowski BA, Younger ST, Hardy DB, Ram R, Huffman KE and Corey DR (2007). Activating gene expression in mammalian cells with promoter-targeted duplex RNAs. Nat Chem Biol 3:166–173 17259978 10.1038/nchembio860

[4] Li L-C, Okino ST, Zhao H, Pookot D, Place RF, Urakami S, Enokida H and Dahiya R (2006). Small dsRNAs induce transcriptional activation in human cells. Proc Natl Acad Sci U S A 103:17337–17342 17085592 10.1073/pnas.0607015103 PMC1859931

[5] Matsui M, Sakurai F, Elbashir S, Foster DJ, Manoharan M and Corey DR (2010). Activation of LDL receptor expression by small RNAs complementary to a noncoding transcript that overlaps the LDLR promoter. Chem Biol 17:1344–1355 21168770 10.1016/j.chembiol.2010.10.009 PMC3071588

[6] Zamecnik PC and Stephenson ML (1978). Inhibition of Rous-sarcoma virus-replication and cell transformation by a specific oligodeoxynucleotide. Proc Natl Acad Sci U S A 75:280–284 75545 10.1073/pnas.75.1.280 PMC411230

[7] Mulamba GB, Hu A, Azad Raana F, Anderson Kevin P and Coen Donald M (1998). Human cytomegalovirus mutant with sequence-dependent resistance to the phosphorothioate oligonucleotide romivirsen (ISIS 2922). Antimicrob Agents Chemother 42:971–973 9559825 10.1128/aac.42.4.971 PMC105584

[8] Merki E, Graham MJ, Mullick AE, Miller ER, Crooke RM, Pitas RE, Witztum JL and Tsimikas S (2008). Antisense oligonucleotide directed to human apolipoprotein B-100 reduces lipoprotein(a) levels and oxidized phospholipids on human apolipoprotein B-100 particles in lipoprotein(a) transgenic mice. Circulation 18:743–753 10.1161/CIRCULATIONAHA.108.786822 18663084

[9] Sharma VK, Sharma RK and Singh SK (2014). Antisense oligonucleotides: modifications and clinical trials. MedChemComm 5:1454–1471

[10] Stein CA and Castanotto D (2017). FDA-approved oligonucleotide therapies in 2017. Mol Ther 25:1069–1075 28366767 10.1016/j.ymthe.2017.03.023 PMC5417833

[11] Watts JK and Corey DR (2012). Silencing disease genes in the laboratory and the clinic. J Pathol 226:365–379 22069063 10.1002/path.2993 PMC3916955

[12] Deleavey GF and Damha MJ (2012). Designing chemically modified oligonucleotides for targeted gene silencing. Chem Biol 19:937–954 22921062 10.1016/j.chembiol.2012.07.011

[13] Eckstein F (1966). Nucleoside phosphorothioates J Am Chem Soc 88:4292–4294 10.1021/ja00718a039 4316997

[14] Eckstein F (2000). Phosphorothioate oligodeoxynucleotides: what is their origin and what is unique about them? Antisense Nucleic Acid Drug Dev 10:117–121 10805163 10.1089/oli.1.2000.10.117

[15] Kumar R, Singh SK, Koshkin AA, Rajwanshi VK, Meldgaard M and Wengel J (1998). The first analogues of LNA (locked nucleic acids): phosphorothioate-LNA and 2′-thio-LNA. Bioorg Med Chem Lett 8:2219–2222 9873516 10.1016/s0960-894x(98)00366-7

[16] Bika S, Nanbu D, Hari Y, Morio M-I, In Y, Ishida T and Imanishi T (1997). Synthesis of 2′O, 4′-C-methyleneuridine and- cytidine. Novel bicyclic nucleosides having a fixed C3-endo sugar puckering. Tetrahedron Lett 38:8735–8738

[17] Kauppinene S, Vester B and Wengel J (2005). Locked nucleic acid (LNA): high affinity targeting of RNA for diagnostics and therapeutics. Drug Discov Today Technol 2:287–290 24981949 10.1016/j.ddtec.2005.08.012 PMC7105916

[18] Vester B and Wengel J (2004). LNA (locked nucleic acid): high-affinity targeting of complementary RNA and DNA. Biochemistry 43:13233–13241 15491130 10.1021/bi0485732

[19] Seth PP, Siwkowski A, Allerson CR, Vasquez G, Lee S, Prakash TP, Kinberger G, Migawa MT, Gaus H, Bhat B and Swayze EE (2008). Design, synthesis and evaluation of constrained methoxyethyl (cMOE) and constrained ethyl (cEt) nucleoside analogs. Nucleic Acids Symp Ser (Oxf) 553–554 10.1093/nass/nrn280 18776499

[20] Wengel J (2008). LNA (Locked Nucleic Acid) and Functionalized LNA: Toward Efficient Gene Targeting, 1st ed. Boca Raton, FL: Taylor and Francis, p 5

[21] Watts JK (2013). Locked nucleic acid: tighter is different. Chem Commun 49:5618–5620 10.1039/c3cc40340h 23682352

[22] Hagedorn PH, Persson R, Funder ED, Albæk N, Diemer SL, Hansen DJ, Møller MR, Papargyri N, Christiansen H, *et al.* (2018). Locked nucleic acid: modality, diversity, and drug discovery. Drug Discov Today 23:101–114 28988994 10.1016/j.drudis.2017.09.018

[23] Horwich MD and Zamore PD (2008). Design and delivery of antisense oligonucleotides to block microRNA function in cultured *Drosophila* and human cells. Nat Protoc 3:1537–1549 18802435 10.1038/nprot.2008.145 PMC2559958

[24] Stenvang J, Petri A, Lindow M, Obad S and Kauppinen S (2012). Inhibition of microRNA function by antimiR oligonucleotides. Silence 3:1 22230293 10.1186/1758-907X-3-1 PMC3306207

[25] Wang Z (2011). The principles of miRNA-masking antisense oligonucleotides technology, in microRNA and cancer. Methods Mol Biol 676:43–49 20931388 10.1007/978-1-60761-863-8_3

[26] Mercatante DR and Kole R (2002). Control of alternative splicing by antisense oligonucleotides as a potential chemotherapy: effects on gene expression. Biochim Biophys Acta 1587:126–132 12084454 10.1016/s0925-4439(02)00075-3

[27] Azani P and Kole R (2003). Therapeutic potential of antisense oligonucleotides as modulators of alternative splicing. J Clin Invest 112:481–486 12925686 10.1172/JCI19547 PMC171400

[28] Siva K, Covello G and Denti MA (2014). Exon-skipping antisense oligonucleotides to correct missplicing in neurogenetic diseases. Nucleic Acid Ther 24:69–86 24506781 10.1089/nat.2013.0461 PMC3922311

[29] Khoo B, Roca X, Chew SL and Krainer AR (2007). Antisense oligonucleotide-induced alternative splicing of the APOB mRNA generates a novel isoform of APOB. BMC Mol Biol 8:3 17233885 10.1186/1471-2199-8-3 PMC1784105

[30] Peacey E, Rodriguez L, Liu Y and Wolfe MS (2012). Targeting a pre-mRNA structure with bipartite antisense molecules modulates tau alternative splicing. Nucleic Acids Res 40:9836–9849 22844088 10.1093/nar/gks710 PMC3479178

[31] Touznik A, Maruyama R, Hosoki K, Echigoya Y and Yokota T (2017). LNA/DNA mixmer-based antisense oligonucleotides correct alternative splicing of the SMN2 gene and restore SMN protein expression in type 1 SMA fibroblasts. Sci Rep 7:3672 28623256 10.1038/s41598-017-03850-2 PMC5473822

[32] Kole R, Krainer AR and Altman S (2012). RNA therapeutics: beyond RNA interference and antisense oligonucleotides. Nat Rev Drug Discov 11:125–140 22262036 10.1038/nrd3625 PMC4743652

[33] Evers MM, Toonen LJA and van Roon-Mom WMC (2015). Antisense oligonucleotides in therapy for neurodegenerative disorders. Adv Drug Deliv Rev 87:90–103 25797014 10.1016/j.addr.2015.03.008

[34] Pendergraff HM, Krishnamurthy PM, Debacker AJ, Moazami MP, Sharma VK, Niitsoo L, Yu Y, Tan YN, Haitchi HM and Watts JK (2017). Locked nucleic acid gapmers and conjugates potently silence ADAM33, an asthma-associated metalloprotease with nuclear-localized mRNA. Mol Ther Nucleic Acids 8:158–168 28918018 10.1016/j.omtn.2017.06.012 PMC5498289

[35] Kurreck J, Wyszko E, Gillen C and Erdmann VA (2002). Design of antisense oligonucleotides stabilized by locked nucleic acids. Nucleic Acids Res 30:1911–1918 11972327 10.1093/nar/30.9.1911 PMC113840

[36] Behlke M (2014). Oral Presentation at the 10th Annual Meeting of the Oligonucleotide Therapeutics Society, San Diego, CA, 102014

[37] Castanotto D, Lin M, Kowolik C, Wang L, Ren X-Q, Soifer HS, Koch T, Rode Hansen B, Oerum H, *et al.* (2015). A cytoplasmic pathway for gapmer antisense oligonucleotide-mediated gene silencing in mammalian cells. Nucleic Acids Res 43:9350–9361 26433227 10.1093/nar/gkv964 PMC4627093

[38] Crooke ST, Wang S, Vickers TA, Shen W and Liang X (2017). Cellular uptake and trafficking of antisense oligonucleotides. Nat Biotechnol 35:230–237 28244996 10.1038/nbt.3779

[39] Liang X, Sun H, Shen W and Crooke ST Identification and characterization of intracellular proteins that bind oligonucleotides with phosphorothioate linkages, Nucleic Acids Res 43:2927–2945 10.1093/nar/gkv143 PMC4357732 25712094

[40] Lorenz P, Baker BF, Bennett CF and Spector DL (1998). Phosphorothioate antisense oligonucleotides induce the formation of nuclear bodies. Mol Biol Cell 9:1007–1023 9571236 10.1091/mbc.9.5.1007 PMC25326

[41] Gonzalez-Barriga A, Nillessen B, Kranzen J, van Kessel IDG, Croes HJE, Aguilera B, de Visser PC, Datson NA, Mulders SAM, *et al.* (2017). Intracellular distribution and nuclear activity of antisense oligonucleotides after unassisted uptake in myoblasts and differentiated myotubes *in vitro*. Nucleic Acid Ther 27:144–158 28375678 10.1089/nat.2016.0641 PMC5467152

[42] Zhao QY, Zhou RZ, Temsamani J, Zhang ZW, Roskey A and Agrawal S (1998). Cellular distribution of phosphorothioate oligonucleotide following intravenous administration in mice. Antisense Nucleic Acid Drug Dev 8:451–458 9918109 10.1089/oli.1.1998.8.451

[43] Moschos SA, Frick M, Taylor B, Turnpenny P, Graves H, Spink KG, Brady K, Lamb D, Collins D, *et al.* (2011). Uptake, efficacy, and systemic distribution of naked, inhaled short interfering RNA (siRNA) and locked nucleic acid (LNA) antisense. Mol Ther 19:2163–2168 21971426 10.1038/mt.2011.206 PMC3242665

[44] Buntz A, Killian T, Schmid D, Seul H, Brinkmann U, Ravn J, Lindholm M, Knoetgen H, Haucke V and Mundigl O (2019). Quantitative fluorescence imaging determines the absolute number of locked nucleic acid oligonucleotide needed for suppression of target gene expression. Nucleic Acid Res 47:953–969 30462278 10.1093/nar/gky1158 PMC6344898

[45] Moreira BG, You Y and Owczarzy R (2015). Cy3 and Cy5 dyes attached to oligonucleotide terminus stabilize DNA duplexes: predictive thermodynamic model. Biophys Chem 198:36–44 25645886 10.1016/j.bpc.2015.01.001

[46] Anderson BJ, Larkin C, Guja K and Schildbach JF (2008). Using fluorophore-labeled oligonucleotides to measure affinities of protein-DNA interactions. Methods Enzymol 450:253–272 19152864 10.1016/S0076-6879(08)03412-5 PMC3051356

[47] Wang K, Rodgers ME, Toptygin D, Munsen VA and Brand L (1998). Fluorescence study of the multiple binding equilibria of the galactose repressor. Biochemistry 37:41–50 9425024 10.1021/bi972004v

[48] Bao G, Rhee WJ and Tsourkas A (2009). Fluorescent probes for live-cell RNA detection. Annu Rev Biomed Eng 11:25–47 19400712 10.1146/annurev-bioeng-061008-124920 PMC2734976

[49] Chen B and Bartlett MG (2012). Determination of therapeutic oligonucleotides using capillary gel electrophoresis. Biomed Chromatogr 26:409–418 21898474 10.1002/bmc.1696

[50] Lin ZJ, Li W and Dai G (2007). Application of LC-MS for quantitative analysis and metabolite identification of therapeutic oligonucleotides. J Pharm Biomed Anal 44:330–341 17339091 10.1016/j.jpba.2007.01.042

[51] Tewary HK and Iversen PL (1997). Qualitative and quantitative measurements of oligonucleotides in gene therapy: part I *in vitro*. J Pharm Biomed Anal 15:857–873 9160252 10.1016/s0731-7085(96)01940-1

[52] McGinnis AC, Chen B and Bartlett MG (2012). Chromatographic methods for the determination of therapeutic oligonucleotides. J Chromatogr B 883–884:76–94 10.1016/j.jchromb.2011.09.007 21945211

[53] Erb R, Leithner K, Bernop-Schnürch A and Oberacher H (2012). Phosphorothioate oligonucleotide quantification by μ-liquid chromatography-mass spectrometry. AAPS J 14 (4) 728–737 22806756 10.1208/s12248-012-9381-2 PMC3475866

[54] Gagnon KT, Li L, Janowski BA and Corey DR (2014). Analysis of nuclear RNA interference (RNAi) in human cells by subcellular fractionation and argonaute loading. Nat Protoc 9:2045–2060 25079428 10.1038/nprot.2014.135 PMC4251768

[55] Straarup EM, Fisker N, Hedtjärn M, Lindholm MW, Rosenbohm C, Aarup V, Hansen H, Ørum H, Hansen JB and Koch T (2010). Short locked nucleic acid antisense oligonucleotides potently reduce apolipoprotein B mRNA and serum cholesterol in mice and non-human primates. Nucleic Acids Res 38:7100–7111 20615897 10.1093/nar/gkq457 PMC2978335

[56] Baghirova S, Hughes BG, Hendzel MJ and Schulz R (2015). Sequential fractionation and isolation of subcellular proteins from tissue or cultured cells. MethodsX 2:440–445 26740924 10.1016/j.mex.2015.11.001 PMC4678309

[57] Cox B and Emili A (2006). Tissue subcellular fractionation and protein extraction for use in mass-spectrometry-based proteomics. Nat Protoc 1:1872–1878 17487171 10.1038/nprot.2006.273

[58] Holden P and Horton WA (2009). Crude subcellular fractionation of cultured mammalian cell lines. BMC Res Notes 2:243 20003239 10.1186/1756-0500-2-243 PMC2802353

[59] Hansen JBR, Orum H, Hansen HF, Straarup EM, Nielsen NF and Hedtjarn M (2013). Short Oligomer Antagonist Compounds for the Modulation of Target mRNA. Santaris Pharma AS

[60] Stein CA, Hansen JB, Lai J, Wu S, Voskresenskiy A, Hog A, Worm J, Hedtjarn M, Souleimanian N, *et al.* (2010). Efficient gene silencing by delivery of locked nucleic acid antisense oligonucleotides, unassisted by transfection reagents. Nucleic Acids Res 38:e3 19854938 10.1093/nar/gkp841 PMC2800216

[61] Varkouhi AK, Scholte M, Storm G and Haisma HJ (2011). Endosomal escape pathways for delivery of biological. J Control Release 151:220–228 21078351 10.1016/j.jconrel.2010.11.004

[62] Erazo-Oliveras A, Muthukrishnan N, Baker R, Wang T-Y and Pellois J-P (2012). Improving the endosomal escape of cell-penetrating peptides and their cargos: strategies and challenges. Pharmaceuticals (Basel) 5:1177–1209 24223492 10.3390/ph5111177 PMC3816665

[63] Lönn P, Kacsinta AD, Cui X, Hamil AS, Kaulich M, Gogoi K and Dowdy SF (2016). Enhancing endosomal escape for intracellular delivery of macromolecular biologic therapeutics. Sci Rep 6:32301 27604151 10.1038/srep32301 PMC5015074

[64] Huber LA, Pfaller K and Vietor I (2003). Organelle proteomics-implications for subcellular fractionation in proteomics. Circ Res 92:962–968 12750306 10.1161/01.RES.0000071748.48338.25

[65] Howell KE, Devaney E and Gruenberg J (1989). Subcellular fractionation of tissue-culture cells. Trends Biochem Sci 14:44–47 2705208 10.1016/0968-0004(89)90040-6

[66] Silhavy TJ, Benson SA and Emr SD (1983). Mechanisms of protein localization. Microbiol Rev 47:313–344 6355805 10.1128/mr.47.3.313-344.1983 PMC281579

[67] Stephens SB and Nicchitta CV (2008). Divergent regulation of protein synthesis in the cytosol and endoplasmic reticulum compartments of mammalian cells. Mol Biol Cell 19:623–632 18077556 10.1091/mbc.E07-07-0677 PMC2230589

[68] Dalton S and Whitbread L (1995). Cell-cycle-regulated nuclear import and export of CDC47, a protein essential for initiation of DNA-replication in budding yeast. Proc Natl Acad Sci U S A 92:2514–2518 7708676 10.1073/pnas.92.7.2514 PMC42248

[69] Mulvey CM, Tudzarova S, Crawford M, Williams GH, Stoeber K and Godovac-Zimmermann J (2013). Subcellular proteomics reveals a role for nucleo-cytoplasmic trafficking at the DNA replication origin activation checkpoint. J Proteome Res 12:1436–1453 23320540 10.1021/pr3010919 PMC4261602

[70] Price DH, Sluder AE and Greenleaf AL (1987). Fractionation of transcription factors for rna polymerase-II from *Drosophila* KC cell nuclear extracts. J Biol Chem 262:3244–3255 3818640

[71] Tilgner H, Knowles DG, Johnson R, Davis CA, Chakrabortty S, Djebali S, Curado J, Snyder M, Gingeras TR and Guigo R (2012). Deep sequencing of subcellular RNA fractions shows splicing to be predominantly co-transcriptional in the human genome but inefficient for IncRNAs. Genome Res 22:1616–1625 22955974 10.1101/gr.134445.111 PMC3431479

[72] Bononi A and Pinton P (2015). Study of PTEN subcellular localization. Methods 77–78:92–103 10.1016/j.ymeth.2014.10.002 PMC4396696 25312582

[73] Singh SK, Kumar R and Wengel J (1998). Synthesis of novel bicyclo [2.2.1] ribonucleosides: 2′-amino-and 2′-thio-LNA monomeric nucleosides. J Org Chem 63:6078–6079 11672223 10.1021/jo9806658

[74] Singh SK, Kumar R and Wengel J (1998). Synthesis of 2′-amino-LNA: a novel conformationally restricted high-affinity oligonucleotide analogue with a handle. J Org Chem 63:10035–10039

[75] Burgers PMJ and Eckstein F (1978). Synthesis of dinucleoside monosphorothioates via addition of sulphur to phosphite trimesters. Tetrahedron Lett 19:3835–3838

[76] Iwamoto N, Butler DCD, Svrzikapa N, Mohapatra S, Zlatev I, Sah DWY, Standley SM, Lu G, Apponi LH, *et al.* (2017). Control of phosphorothioate stereochemistry substantially increases the efficacy of antisense oligonucleotides. Nat Biotechnol 35:845–851 28829437 10.1038/nbt.3948

