# Supplementary material for: Nuclear and Cytoplasmatic Quantification of Unconjugated, Label-Free Locked Nucleic Acid Oligonucleotides
Source: Nucleic Acid Ther. 2020 Jan 28;30(1):4–13. doi: 10.1089/nat.2019.0810 (PMC6987631; doi:10.1089/nat.2019.0810)
Supplement: Supplemental data [file Supp_Fig1.pdf]

Supplementary Data

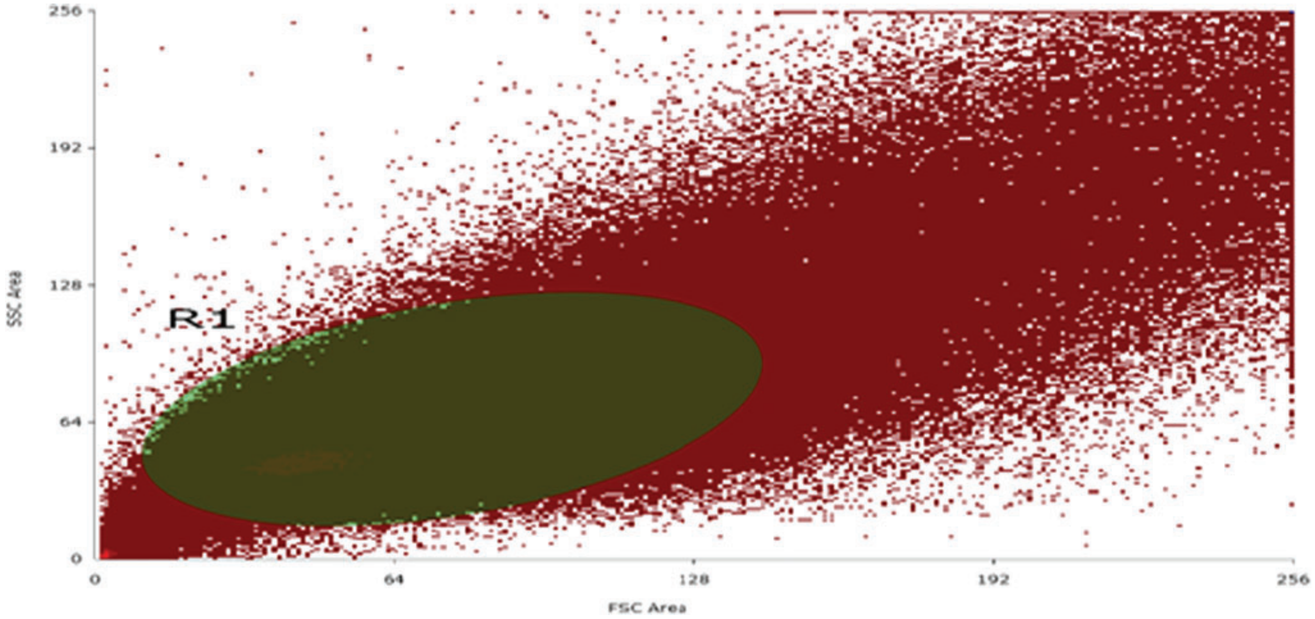

SUPPLEMENTARY FIG S1. Representative image from the BioRad cell sorter showing the section of nuclei that were selected for further experiments. The selected circle represents ~70%–85% of the initial nuclei input.
