# Supplementary material for: Nuclear and Cytoplasmatic Quantification of Unconjugated, Label-Free Locked Nucleic Acid Oligonucleotides
Source: Nucleic Acid Ther. 2020 Jan 28;30(1):4–13. doi: 10.1089/nat.2019.0810 (PMC6987631; doi:10.1089/nat.2019.0810)
Supplement: Supplemental data [file Supp_Table1.pdf]

Supplementary Table S1. List of Compound Design and Nucleobase Sequences Used for This Work. Uppercase: Locked Nucleic Acid; Lowercase: DNA; Subscript s (s): Phosphorothioate

**LNA**	**Target**	**sequence**
LNA1	HIF-1a	$G_sC_sa_sa_sg_sc_sa_st_sc_sc_st_sG_sT$
LNA2	BCL2	$A_sG_sT_st_sa_st_sa_sa_st_sc_sc_sa_sG_sC_sT$
LNA3	Malat1	$G_sA_sG_st_st_sa_sc_st_st_sg_sc_sc_sa_sA_sC_sT$
LNA4	Cers2	$T_sT_sG_st_st_sa_st_st_sg_sa_sg_sg_sa_sT_sG_sG$
LNA5	Cers2	$A_sG_sC_st_st_st_sg_sg_sc_sa_sg_sa_sa_sT_sG_sA$
LNA6	PTEN	$T_sC_sA_sc_st_st_sa_sg_sc_sc_sa_st_st_sG_sG_sT$
