# Supplementary material for: Nuclear and Cytoplasmatic Quantification of Unconjugated, Label-Free Locked Nucleic Acid Oligonucleotides
Source: Nucleic Acid Ther. 2020 Jan 28;30(1):4–13. doi: 10.1089/nat.2019.0810 (PMC6987631; doi:10.1089/nat.2019.0810)
Supplement: Supplemental data [file Supp_Fig2.pdf]

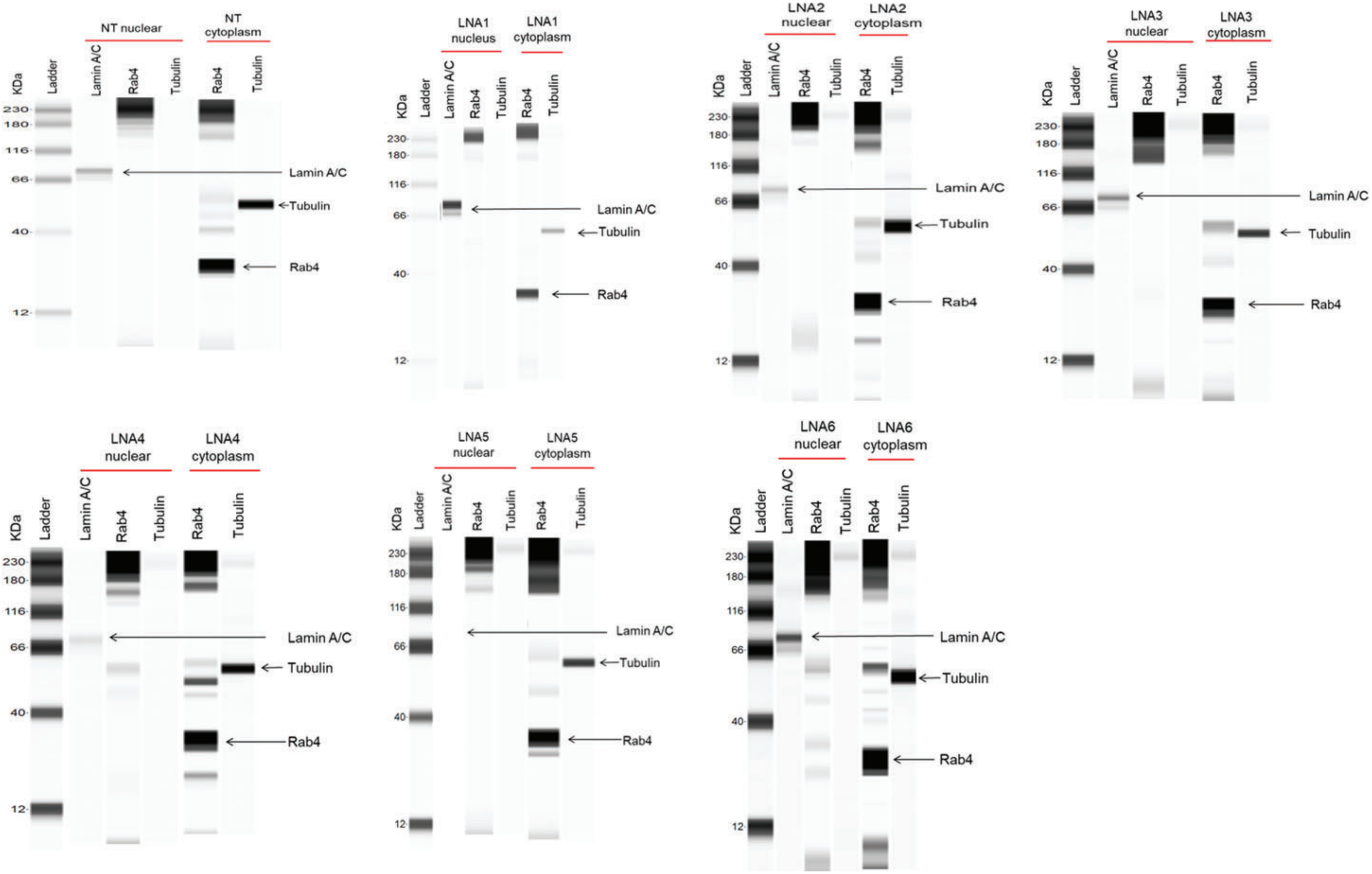


**SUPPLEMENTARY FIG S2.** WES ProteinSimple analysis showing the purity of our nuclear fractions. 0.2 mg/mL of protein was added to each well.
