# Supplementary material for: Nuclear and Cytoplasmatic Quantification of Unconjugated, Label-Free Locked Nucleic Acid Oligonucleotides
Source: Nucleic Acid Ther. 2020 Jan 28;30(1):4–13. doi: 10.1089/nat.2019.0810 (PMC6987631; doi:10.1089/nat.2019.0810)
Supplement: Supplemental data [file Supp_Table2.pdf]

Supplementary Table S2. Enzyme-Linked Immunosorbent Assay Minimum Detection Limits for the Probes Used in This Study.

LNA	Target	Minimum detection limit	Minimum measured value
LNA1	Hif-1-alpha	0.15 pmol/mL	0.39 pmol/mL
LNA2	BCL2	0.35 pmol/mL	2.96 pmol/mL
LNA3	Malat1	0.18 pmol/mL	3.72 pmol/mL
LNA4	Cers2	0.07 pmol/mL	8.98 pmol/mL
LNA5	Cers2	0.03 pmol/mL	9.55 pmol/mL
LNA6	PTEN	0.11 pmol/mL	5.10 pmol/mL

All our data were over the limit of detection for their appropriate assays.
